# Preferred problem solving and decision-making role in fertility treatment among women following an unsuccessful in vitro fertilization cycle

**DOI:** 10.1186/s12905-019-0856-5

**Published:** 2019-12-05

**Authors:** Celia Hoi Yan Chan, Bobo Hi Po Lau, Michelle Yi Jun Tam, Ernest Hung Yu Ng

**Affiliations:** 1 Department of Social Work and Social Administration, The University of Hong Kong, The Jockey Club Tower, Hong Kong, Hong Kong; 2 Department of Obstetrics and Gynecology, The University of Hong Kong, Hong Kong, Hong Kong

**Keywords:** Problem solving, Decision-making, IVF, Patient-centred care, Spousal relationship

## Abstract

**Background:**

While the literature on healthcare decision-making has long focused on doctor-patient interaction, fertility treatment is an exception, characterized by a triangular interplay between the doctor, the woman and her partner. This study examined treatment decision-making preferences of women undergoing in vitro fertilization (IVF) treatment, following an unsuccessful IVF cycle, especially their preferred level of doctor and spousal involvement.

**Methods:**

A cross-sectional survey was conducted with 246 Chinese women undergoing IVF recruited from an assisted reproduction clinic of a university-affiliated hospital in Hong Kong. Data collection was conducted between January 2014 and August 2015.

**Results:**

Most participants preferred sharing the decision-making tasks with their doctors (92%). In the doctor-patient relationship, passive roles were associated with higher marital satisfaction, presence of religious affiliation and secondary infertility, while autonomous roles were related to female-factor infertility. Fifty-two percent of participants anticipated sharing decision-making, while 46% preferred handing over the decision to their husbands. Preference for a passive rather than a shared role in the spousal relationship was related to a higher husband’s age, greater marital satisfaction and higher anxiety.

**Conclusions:**

In brief, women tended to prefer sharing decision-making tasks with their doctor as well as actively engaging their partner in making decisions about fertility treatment. This study adds to our understanding of women’s role preference and level of involvement in infertility treatment decision-making by providing quantitative evidence from women’s experience. It highlights the importance of healthcare professionals in facilitating shared decision-making among couples.

## Background

Fertility treatment is characterized by its open-ended nature. Deciding on treatment options, including continuation and termination, is an emotionally-charged but ambivalent process. Women who have recently experienced an unsuccessful fertility treatment cycle often find themselves at a crossroads; pursuing the next treatment instils hope, but also requires re-experiencing treatment anxiety, whereas treatment termination requires enormous courage to admit such hope will no longer be realized [1], but could also provide relief from a long struggle [2].

Previous studies have revealed that during the decision-making process in fertility treatment, women often assume personal responsibility for treatment decisions [2–4]. Most women expect honest and personalized medical information from healthcare professionals, even when such information is unfavourable (e.g., low chances of success) [2, 5, 6]. Rauprich and colleagues [5] reported that healthcare professionals also assume that the ultimate decision rests with the woman, even where there is a high likelihood of treatment failure. To honour patients’ rights to know and participate in treatment planning, the model of shared decision-making endorses active patient involvement as a collaborating partner in clarifying acceptable options and agreeing a desirable course of action [7]. This model is appropriate where there is more than one reasonable course of action, including invasive treatment, supportive care or treatment withdrawal, and no single option is the best for everyone [8]. While patients’ active participation has generally been associated with improved treatment satisfaction and clinical outcomes in other disease contexts [9–11], in practice, most women seem to be poorly-informed about the likelihood of treatment success, side-effects and risks of treatment (e.g., multiple pregnancies), emotional distress during treatment, circumstances for exiting treatment, and how their desire for a child may impact rational decision-making in fertility treatment [2, 3, 5]. Hence, there does not seem to be any golden rule about how extensively patients should be engaged in treatment decisions in fertility treatment.

It is imperative to clarify patients’ preferences for medical information and participation in decision-making [12] since the match between preferred and perceived levels of participation impacts post-treatment adjustment and quality of life [13, 14] . Brom and colleagues [15] discovered that congruence between patients’ preferred and perceived participation in medical decision-making occurred in only 60% of cases, and that in cases of incongruence the proportions of patients who preferred more involvement and those preferring less involvement were comparable. Engaging patients in accordance with their preferred role in treatment decision-making was found to be beneficial, especially in instances associated with intense emotions and demanding treatment [16].

In most chronic conditions (e.g., cancer, fractures), shared decision-making applies within a bi-partite patient-doctor relationship. However, in fertility care, the decision to initiate, continue or terminate treatment is characterized by a unique interplay between the doctor, the woman and her partner [4, 17]. Although men tend to enter fertility treatment for their partners and women both for themselves and their partners [18], the decision to initiate fertility treatment is based on consensus between the couple about having a child and sustained by their commitment to endure the treatment together [2, 4]. Among heterosexual couples it is a mutual project where both partners have to constantly reflect and deliberate on what is important to them, whether the current options are acceptable for actualizing their fertility goals, and subsequently arriving at a treatment decision acceptable to both [19].

Husbands often experience enormous stress during fertility treatment decision-making and feel a lack of control and engagement in the process [20, 21]. Olafsdottir and colleagues [4] found that husbands tend to perceive their wife as the one pushing forward with the decision to undergo treatment, and that since most treatment procedures are conducted on their wife, they are not in a position to pressurize her to undergo further treatment despite having a comparable desire for a child. Many husbands also feel helpless witnessing their wife experiencing the pain and stress of treatment while there is little they can do except provide support and impose a “rational veto” to end further treatment when necessary [22]. However, terminating treatment is a precarious move, as often neither of the couple wants to be seen as “admitting defeat” [2]. Our current understanding of what wives, being at the centre of fertility treatment, expect from the engagement of their doctor and husband at times of major treatment decision is still limited. Such knowledge is, however, imperative for facilitating the communication of medical information and subsequent fertility treatment decision-making.

### Setting for the current study

In traditional Chinese culture, childbearing is an important task for married couples. Being childless is regarded as one of the most unfilial acts. A male heir serves not only to provide for his aging parents (and grandparents), but also to continue the paternal bloodline and inherit the family’s wealth. Married couples therefore often face significant pressures from their families to produce a male heir. Although in contemporary Chinese culture less importance is placed on the child’s gender, many subscribe to the idea that a child completes a family and see childbearing as a significant developmental milestone for a married couple [23]. Thus, being childless often stigmatizes married couples [24]. Fertility treatment provides an avenue for childless couples to attain their fertility goal. In 2018, 5055 in vitro fertilization (IVF) cycles, the most common form of assisted reproduction technologies (ART), were conducted in Hong Kong [25]. In this study, we examined the preference of participation in treatment decisions among Chinese women undergoing IVF).

To allow within-subject comparison across different health contexts, our study employed vignettes on both a general health issue and fertility treatment. We also compared our findings for the fertility treatment vignette with those of a Canadian sample of 428 infertile women [16]. To understand the couple dynamics involved in making fertility treatment decisions, we evaluated the women’s preference for both their doctors’ and their husband’s participation.

## Methods

### Study population

Women who did not conceive following an IVF cycle were recruited from an assisted reproduction clinic in a university-affiliated hospital in Hong Kong. Women were excluded from the study if they were unable to: (i) communicate in Chinese, (ii) attend the consultation session; or (iii) provide written informed consent.

### Procedure

Eligible participants were approached by a research assistant after being informed of a negative urine pregnancy test and before they attended a follow-up consultation session with the doctor. They were introduced to the study and given a cover letter and informed consent form. Those who agreed to participate were asked to complete the questionnaires, which included items about their preference in treatment decision-making, attitudes and experience of fertility treatment, well-being, reproductive history, and demographic details. Data collection was undertaken between January 2014 and August 2015.

### Measurements

#### Preferred role of participation in decision-making

**Fig. 1:**
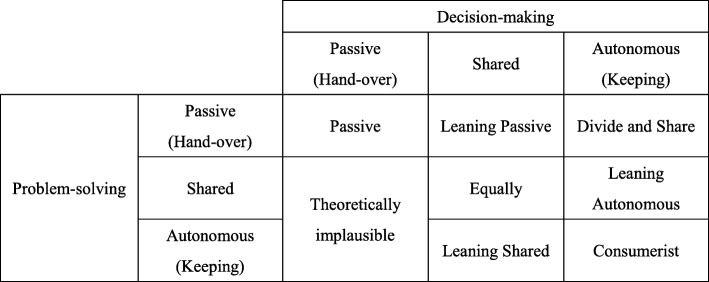
Preferred roles in treatment decision according to Deber and colleagues (Deber, 1994 [26]; Deber & Baumann, 1992 [27]; Deber et al., 1996 [28])

To measure women’s preference for participation in treatment decisions, we adopted Deber and colleagues’ Problem Solving-Decision Making (PSDM) model [16]. This distinguishes two types of decision tasks: (i) Problem-solving (PS), where there is one correct answer and determination requires expertise rather than idiosyncratic preference, and (ii) Decision-making (DM), which involves weighing the relative importance of potential outcomes based on both factual knowledge and personal preference. An individual’s preferred level of participation in PS and DM can be categorized as – (i) handing over the task to another person, (ii) sharing the task with another person, and (iii) undertaking the task oneself. Individuals’ preferred levels of participation in PS and DM together place them in one of the following preferred roles in a 3 × 3 rubric (Fig. 1). According to the PSDM model, Shared roles include (i) Leaning passive, (ii) Shared equally, (iii) Divide and share, and (iv) Leaning autonomous, whereas Autonomous roles comprise (i) Leaning shared and (ii) Autonomous/consumerist. Sharing or keeping PS but handing over DM is considered theoretically implausible [16].

The PSDM scale [16] contains four questions on PS (e.g. “Who should decide what the treatment options are?”) and two questions on DM (e.g. “Given all the information about risks and benefits of the possible treatments, who should decide which treatment options should be selected?”). Participants were first presented with a general health vignette (“Suppose you had mild chest pains for three days and decided that you should visit your doctor about this”) and then a vignette related to fertility treatment (“Suppose you and your husband were unable to conceive after one year of unprotected sex and decided to visit your doctor about this”).

In the chest pain vignette, participants were instructed to indicate their choice on a 5-point scale with 1 (doctor alone), 2 (mostly the doctor), 3 (both equally), 4 (mostly me) and5 (me alone) for each question. Mean scores were computed separately for PS (α = .76) and DM (α = .79). Participants were then assigned to one of three categories for PS and DM: “Hand over” (dimension mean score < 3); “Share” (mean score between 3 and 3.99); and “Keep” (mean score ≥ 4). These classifications were then used to place participants in one of the following preferred roles: Passive, Shared (Leaning passive, Shared equally, Divide and share, Leaning autonomous), Autonomous (Leaning shared, Autonomous/consumerist) or Theoretically implausible combinations (see Fig. 1).

In the fertility treatment vignette, to measure wives’ preferred role of participation within the spousal relationship, a third decision-maker category, “husband”, was introduced in addition to “doctor” and “me”. Participants were asked to indicate their choice from 0 (no involvement at all) to 4 (mostly involved) for doctors (PS: α = .81; DM: α = .82), husbands (PS: α = .73; DM: α = .77), and themselves (PS: α = .83; DM: α = .83) on each question. To align our scoring algorithm with that of Deber and colleagues [16] the following recoding was conducted for each question: “Doctor (or husband) alone” of Deber and colleagues’ [16] scale was indicated if the score for doctor (or husband) was more than 1 point higher than that of the participant’s. “Mostly doctor (or husband)” was indicated if the doctor’s (or husband’s) score was only 1 point more than the participant’s. “Both equally” was indicated by identical scores given to the doctor (or husband) and the participant. “Mostly me” was indicated by 1 point higher for the participant than the doctor (or husband). “Me alone” was indicated by higher than 1 point more for the participant than the doctor (or husband). Where neither party scored higher than 2 on the scale running from 0 to 4, but one had a score more than 1 point higher than another, “mostly me (or doctor or husband)” was assigned. (A conversion table is included in the Additional file 1). Then, again, the 5-point scale from the chest pain vignette (i.e., 1 (doctor or husband alone) to 5 (me alone)) was mapped onto the recoded response for each question, followed by calculating the mean scores of PS and DM which show the preference for “hand over”, “shared” and “keep” for the two tasks in the doctor-patient and the spousal relationship. Lastly, the preferred role of participation (Passive, Shared, Autonomous, Theoretically implausible) was determined in the same way as in the chest pain vignette.

The Canadian sample for comparison was from Stewart and colleagues [3] who used the PSDM scale to evaluate the participation preferences of women undergoing fertility treatment. Deber et al. (2007) [16] used the raw data from this study and analysed 454 women’s participation preferences. Regarding the PSDM scale, responses from 26 women were unavailable, leaving 428 responses available for analysis.

#### Demographic, clinical and well-being characteristics

Demographic characteristics (age, education, employment status, religious affiliation, husband’s age, years of marriage) and clinical information (years of infertility, type of infertility, presence of a living child, cause of infertility, duration of ART) were obtained for all participants. Well-being indicators including health-related quality of life, anxiety, depression and marital satisfaction were measured by the Chinese versions of Fertility-related Quality of life (FertiQOL) [29], the Hospital Anxiety and Depression Scale (HADS) [30] and the Kansas Marital Satisfaction Scale (KMSS) [31], respectively. The 36-item FertiQOL (α = .918) comprises two scales: Core (24 items; α = .92) and Treatment (10 items; α = .79). Higher scores indicate better quality of life. HADS contains seven items each on depression (α = .78) and anxiety (α = .87), with higher scores indicating greater distress. KMSS contains three items with higher scores representing higher marital satisfaction (α = .94).

### Statistical analysis

Chi-square statistics were used to compare chest pain and fertility treatment preferences of our sample and with their Canadian counterparts. The associations between role preference and participants’ demographic, clinical and well-being characteristics were explored with multiple logistic regressions. The analysis was conducted using SPSS (version 25.0).

## Results

Three hundred and eighty-nine women who fulfilled the inclusion criteria were identified through the patient record system. After initial screening, 71 women were excluded because they met one or more exclusion criterion. Of the 318 women who were subsequently approached, 72 did not provide informed consent, resulting in 246 who completed the questionnaire. The response rate was 63%.

Table 1 reports participants’ demographic and clinical characteristics. The mean age of participants was 37.2 years (standard deviation 3.4; range: 27–46). Slightly more than half of the sample received tertiary education (60%) and nearly three-quarters (73%) were in full-time employment (73%). Nearly two-thirds (64%) reported no religious affiliation. They had been married for an average of 7.3 years (standard deviation 3.8) and their husband’s mean age was 40.2 years (standard deviation 5.6). The average duration of infertility was 4.0 years (standard deviation 2.5), with slightly more than half diagnosed with primary infertility (53%). Female factor infertility was diagnosed in 30% and male factor infertility in 29% of the cases. More than half had undergone previous ART procedures (average number of years spent in ART treatment = 2.8). Forty-two percent indicated the current procedure as their first failure, and 55% involved a fresh embryo transfer cycle.

**Table 1:** Demographic and clinical characteristics of participants (*N* = 246)

Characteristics	n	%
Age of woman (years)		
Mean / standard deviation (range)	37.2 / 3.4 (27–46)	
30 or below	6	2
31–35	67	27
36–40	134	55
40+	39	16
Education Level		
Secondary education or below	99	40
Tertiary education	147	60
Employment		
Employed (Part-time)	20	8
Employed (Full-time)	180	73
Unemployed	41	17
Others	5	2
Religion		
None	158	64
Ancestral Worship	13	5
Catholic	5	2
Protestant / Non-denominational	46	19
Buddhist	22	9
Others	2	1
Husband’s Age (years)		
Mean / standard deviation (range)	40.2 / 5.6 (29–63)	
30 or below	3	1
31–35	46	19
36–40	96	39
41–45	61	25
45+	40	16
Duration of Marriage (years)		
Mean / standard deviation (range)	7.3 / 3.8 (1–19)	
5 or below	92	37
6–10	113	46
10+	41	17
Duration of infertility (years)		
Mean / standard deviation (range)	4.0 / 2.5 (1–14)	
Type of infertility		
Primary	131	53
Secondary	115	47
No. of live births		
0	208	85
1 or more	38	15
No. of pregnancy loss		
Mean / standard deviation (range)	0.7 / 1.1 (0–6)	
0	150	61
1	56	23
2 or more	40	16
Causes of infertility		
Unexplained	53	22
Male factor only	70	28
Female factor only	74	30
Mixed factor	49	20
Duration of ART treatment (years)		
Mean / standard deviation (range)	2.8 / 2.1 (0.3–8.9)	
Less than a year	49	20
1 to 3 years	133	54
4 to 5 years	36	15
6 years or more	28	11
Unsuccessful treatment procedure at T_0_		
Fresh embryo transfer (ET)	134	55
Frozen embryo transfer (FET)	111	45
No. of previous failed IVF cycles		
Mean / standard deviation (range)	1.1 / 1.4 (0–8)	
0	102	41
1	80	33
2	36	15
3 or more	28	11
Remaining frozen embryos?		
Yes	110	45
No	133	55

### Decision-making participation preferences

Table 2 reports participants’ decision-making participation preferences. In the fertility treatment vignette, mostparticipants opted for Shared (92%), with a majority choosing Shared equally (74%), while only a minority preferred Passive (2%) in the doctor-patient relationship. However, in the chest pain vignette, a more equal distribution between Shared and Passive roles was evident, with 42% endorsing Leaning passive (Shared) and 41% endorsing Passive. Extremely few participants opted for Autonomous in either scenario (chest pain: 0.4%; fertility treatment: 4%) or the Theoretically implausible combinations (chest pain: 2%; fertility treatment: 3%).

**Table 2:** Categorization of preferred roles in treatment decisions

Study Sample	Current sample (doctor-patient) n (%)	Current sample (spousal) n (%)	Current sample n (%)	Canadian women in IVF treatment^b^ (%)
Used vignette	Fertility treatment	Fertility treatment	Chest pain	Fertility treatment^a^
Passive (hand over PS & DM)	5 (2)	114 (46)	100 (41)	10
Shared - Leaning passive	5 (2)	72 (29)	102 (41)	37
Shared – Equally	183 (74)	8 (3)	16 (7)	9
Shared - Leaning autonomous	35 (14)	5 (2)	3 (1)	8
Shared - Divide and share	2 (1)	44 (18)	19 (8)	35
Shared - total	225 (91)	129 (52)	140 (57)	89
Autonomous - Leaning shared	2 (1)	0	0	0
Autonomous – Consumerist	7 (3)	1 (0)	1 (0)	
Autonomous - total	9 (4)	1 (0)	1 (0)	0
Theoretically implausible (shared/keep PS and hand over DM)	7 (3)	2 (1)	5 (2)	1
N	246 (100)	246 (100)	246 (100)	428

^a^No. frequency data were available from Deber et al., 2007 [16]

^b^Data from Deber et al., 2007 [16]

Due to the small cell sizes, in the subsequent analyses we combined the four roles under Shared (Leaning passive, Shared equally, Leaning autonomous, Divide and share) and excluded Autonomous (accounting for only 0.4% in the chest pain vignette and 0% in the Canadian fertility study) and Theoretically implausible (accounting for only 2% in the chest pain vignette and less than 1% in Canadian fertility study).

There is a significant difference in the distribution of Passive versus Shared roles between the chest pain and the fertility treatment vignettes, χ^2^(1) = 105.58, *p* = < 0.0001. Significant differences in the distribution of Shared and Passive roles were also found between the Hong Kong and Canadian samples on the fertility treatment vignette, χ^2^(1) = 5.26, *p* = 0.022.

In other words, Shared roles accounted for a significantly greater proportion than Passive in the fertility treatment vignette than in the chest pain vignette in the Hong Kong sample, and in the Hong Kong sample than in the Canadian sample for the fertility treatment vignette.

Within the couple, about half of the sample (46%) preferred to hand over both PS and DM to their husband,. although slightly more than half (52%) opted to share the tasks with their husband. Autonomous (0.4%) and Theoretically implausible (0.8%) remained a minority choice. The distribution of Passive versus Shared was significantly different in the doctor-patient relationship and spousal relationship, χ^2^(1) = 125.61, *p* = < 0.0001. Passive was significantly more frequently endorsed in the spousal relationship than in the doctor-patient relationship.

### Factors associated with decision-making participation preferences in the doctor-patient relationship

Due to the extreme distribution of cell sizes in the doctor-patient relationship in the fertility treatment vignette based on Deber and colleagues’ categorization [16], we re-grouped the preferences by combining Passive and Leaning passive to form a “Passive and leaning passive” category, and combining Leaning autonomous, Divide and share and the two Autonomous roles to create an “Autonomous and leaning autonomous” category. We excluded Theoretically implausible from the following analysis because of insufficient cell size (3%) and because these combinations are not conceptually anticipated in Deber and colleagues’ model. Therefore, there were three dependent categories in the nominal logistic regression – Equally (Coded as the reference category as it is the largest; 74%), Passive and leaning passive (4%), and Autonomous and leaning autonomous (19%). The resultant model was significant, Χ^2^(36) =65.08, *p* = 0.002, with a Nagelkerke R-square of 0.33 (Table 3). Compared with participants who endorsed Shared equally, participants who opted for Passive and leaning passive reported higher marital satisfaction, were more likely to have a religious affiliation and were diagnosed with secondary rather than primary infertility. Furthermore, relative to participants who preferred Shared equally, the cause of infertility among participants choosing Autonomous and leaning autonomous was more likely to be female-factor than mixed-factor. Other factors were non-significant.

**Table 3:** Results of nominal logistic regression on preferred roles in treatment decisions in doctor-patient relationship (*N* = 236)

Variables	Passive and leaning passive vs Shared equally			Autonomous and leaning autonomous vs Shared equally		
	OR	*p*-value	95%CI of OR	OR	*p*-value	95%CI of OR
Higher woman’s age in years	0.77	0.149	0.55–1.10	1.02	0.832	0.89–1.16
Having tertiary education (Ref: Secondary education or less)	2.37	0.504	0.19–29.90	0.89	0.781	0.40–1.98
Having full-time occupation (Ref: Absence)	0.86	0.886	0.10–7.18	1.55	0.352	0.62–3.91
Having a religious affiliation (Ref: Absence)	7.79	0.048	1.02–59.50	1.98	0.072	0.94–4.17
Higher husband’s age in years	1.19	0.086	0.98–1.45	1.04	0.298	0.96–1.13
Longer years of marriage	0.81	0.287	0.55–1.19	1.00	0.942	0.89–1.13
Longer years of infertility	1.40	0.194	0.84–2.33	0.91	0.241	0.77–1.07
Secondary infertility (Ref: Primary infertility)	14.45	0.033	1.25–167.59	1.54	0.321	0.66–3.64
Presence of live birth (Ref: Absence)	0.18	0.308	0.00–4.93	0.64	0.432	0.21–1.96
Cause of infertility – Female factor only (Ref: Mixed infertility)	2.55	0.504	0.16–39.46	3.13	0.045	1.03–9.51
Cause of infertility – Male factor only^a^ (Ref: Mixed infertility)				1.95	0.258	0.61–6.22
Cause of infertility – Unexplained (Ref: Mixed infertility)	2.58	0.539	0.13–53.25	1.64	0.433	0.48–5.70
Longer duration of ART in years	1.73	0.061	0.98–3.01	1.17	0.120	0.96–1.42
Higher fertility-related quality of life – Core	0.97	0.610	0.87–1.08	0.98	0.332	0.95–1.02
Higher fertility-related quality of life – Treatment	0.94	0.257	0.85–1.04	0.98	0.330	0..95–1.02
Higher marital satisfaction	1.73	0.036	1.04–2.89	0.90	0.098	0.78–1.02
Higher anxiety	0.88	0.378	0.65–1.18	1.05	0.430	0.93–1.18
Higher depression	1.12	0.624	0.71–1.77	0.90	0.206	0.40–1.98

Note. Shared equally = reference category for the dependent variable. *OR* Odds ratio, *CI* Confidence interval, *ART* Assisted reproductive technology

^a^No participants in the Passive and leaning passive category suffered from male-factor infertility. Therefore, the odds ratio and the corresponding *p*-value are not available

### Factors associated with decision-making participation preferences in the spousal relationship

As the re-grouping of roles did not yield a more balanced distribution of cell sizes (Passive and leaning passive = 76%, Equally = 3%, Autonomous and leaning autonomous = 20%), and since most participants (99%) opted for either Passive or Shared roles, we excluded Autonomous roles in the following analysis and followed the grouping of Deber and colleagues [16] for Passive and Shared roles. A binary logistic regression was conducted to explore the associations between demographic, clinical and well-being factors and the preference for Passive versus Shared (coded as the reference category as it is the largest; 52%) roles. The model was significant, Χ^2^(18) = 36.24, *p* = 0.007, with a Nagelkerke R-square of 0.19 (Table 4). Preference for Passive (versus Shared) was related to a higher husband’s age, greater marital satisfaction and more anxiety. Other factors were non-significant.

**Table 4:** Results of binary logistic regression with preferences of Shared versus Passive roles in treatment decisions in spousal relationship (*N* = 246)

Variables	OR	*P*-value	95%CI of OR
Higher woman’s age in years	0.92	0.118	0.83–1.02
Having tertiary education (Ref: Secondary education or less)	0.58	0.079	0.31–1.06
Having full-time occupation (Ref: Absence)	0.84	0.604	0.42–1.65
Having a religious affiliation (Ref: Absence)	0.94	0.844	0.52–1.70
Higher husband’s age in years	1.09	0.012	1.02–1.16
Longer years of marriage	1.05	0.298	0.96–1.16
Longer years of infertility	1.00	0.966	0.88–1.14
Secondary infertility (Ref: Primary infertility)	0.79	0.499	0.40–1.57
Presence of live birth (Ref: Absence)	0.99	0.977	0.39–2.48
Cause of infertility – Female-factor (Ref: Mixed infertility)	0.71	0.408	0.31–1.60
Cause of infertility – Male-factor (Ref: Mixed infertility)	0.62	0.253	0.27–1.41
Cause of infertility – Unexplained (Ref: Mixed infertility)	0.82	0.659	0.32–2.01
Longer duration of ART in years	0.85	0.057	0.73–1.01
Higher fertility-related quality of life – Core	0.99	0.460	0.96–1.02
Higher fertility-related quality of life - Treatment	1.02	0.214	0.99–1.04
Higher marital satisfaction	1.21	0.001	1.08–1.36
Higher anxiety	1.13	0.012	1.03–1.25
Higher depression	0.92	0.220	0.80–1.05

Note. Shared role = reference category for the dependent variable. *OR* Odds ratio, *CI* Confidence interval, *ART* Assisted reproductive technology

## Discussion

This study examined fertility treatment decision-making participation preferences among Chinese women following a recent unsuccessful IVF cycle. Most participants prefer to share decision-making than handing over this task to their doctors or make decisions themselves. In agreement with Deber and colleagues [16] the preference for sharing rather than handing over decision-making tasks was higher for a specific health condition (i.e. fertility treatment) than a general health condition (i.e. mild chest pain). Previous studies have reported that couples experiencing infertility are keen to search for treatment-related information and share this with their healthcare professionals in order to maximizing the chances of successful treatment [4, 32]. As our participants were not completely new to IVF, they were likely to be more knowledgeable about fertility treatment than a dubious chest pain.

Despite the greater power distance between patients and healthcare professionals that characterises Chinese culture compared to Canadian culture [33], we documented a greater preference for Shared roles (and a lower preference for a Passive role) in our Hong Kong sample than in the Canadian study. In fact, no Canadian participants chose Autonomous roles, while a minority of our participants did so. While a direct comparison was not feasible, our participants were in fertility treatment for an average of 4.0 years (+/− 2.5) and had completed at least one IVF cycle, while their Canadian counterparts were in treatment for 2.3 years (+/− 2.6) only. The longer duration of fertility treatment may have led to greater self-efficacy in sharing treatment decision tasks. However, the effect of previous clinical experience (e.g. years of infertility, years of ART) did not result in a significant difference in preferences in the Hong Kong sample, after controlling for other demographic, clinical and well-being factors. Nonetheless, our findings serve to demonstrate the variety of possibilities regarding cultural differences in healthcare decision-making and the multi-factorial nature of patients’ preferences.

Our findings reveal several demographic and clinical factors related to decision-making participation preferences in the doctor-patient relationship. First, in agreement with previous studies, participants with a religious affiliation tended to be more passive than those without a religious affiliation, possibly due to a greater tendency to trust authorities [34]. Due to the small cell sizes and the lack of existing literature on the effect of different religions on the fertility experience of the Chinese population, by the principle of parsimony, we only dichotomized the sample into those who reported and those who did not report a religious affiliation. However, future research may explore the nuances of the impact of different religions on the experience of fertility treatment among East Asian patients. Passivity in treatment decision-making was also related to the diagnosis of secondary infertility, rather than primary infertility. Participants diagnosed with secondary infertility may have greater difficulties making sense of their current fertility problems as they have previously achieved a clinical pregnancy, irrespective of the outcome (live birth, ectopic pregnancy, or miscarriage). Hence, with greater uncertainty and complications regarding their reproductive potential, they may exhibit a higher tendency to rely on healthcare professionals for treatment decision-making. Likewise, relative to women confronting infertility of mixed causation, women with female factor only infertility tended to be more autonomous in fertility treatment decision-making. This greater autonomy could have been encouraged by the greater certainty of attributing the cause of infertility to oneself, and subsequently greater perceived responsibility for the condition and its treatment.

Nonetheless, in spousal relationships, nearly half of our sample preferred to hand over both PS and DM to their husband. The percentage of participants who preferred to share decision-making tasks dropped from 92% in the doctor-patient relationship to 52% in the spousal relationship. Being autonomous, however, remained a minority choice. The options postulated to be theoretically implausible by Deber and colleagues [16] were rare in the spousal context.

Several factors were related to the tendency to hand over rather than share decision-making tasks in the spousal relationship. Having controlled for the woman’s age, a higher husband’s age was related to a greater tendency to hand over rather than share decision-making tasks. The larger spousal age gap, especially when the husband is the older spouse, may have enlarged the power imbalance between a couple, leading to a greater preponderance of the husband’s view as regards infertility and its treatment. This could be particularly pertinent in Chinese culture where the child bears only the paternal family surname and bloodline. Higher anxiety in women was also related to a greater tendency to entrust the decision-making tasks to their husband. Anxiety may have fuelled a woman’s wish for her husband to shoulder the psychological burdens of decision-making.

In contrast to the shared decision-making model [7], according to which patients enjoy better adjustment with active engagement in the treatment decision-making process, in this study Passive roles in both the doctor-patient and spousal relationships were related to higher marital satisfaction. Our study cannot clarify the direction of causality between marital satisfaction and decision-making participation preferences. However, several explanations are possible. First, entrusting the tasks to a knowledgeable outsider, such as a doctor, may avoid relational conflicts, especially when the couple are divided in their views over infertility and its treatment. Active involvement or even handing over key tasks in treatment decision-making to the husband may foster mutual trust and commitment and enhance relational quality in fertility treatment where husbands are often side-lined [35]. Hence, handing over the decision-making tasks to doctors and husbands may enhance relational quality. On the other hand, higher relational quality may increase the tendency to hand over decision-making tasks to doctors or husbands. Inviting the husband to PS and DM requires pre-established trust that the couple are on the same page and share similar views about treatment.

Our participants had experienced a recent unsuccessful IVF cycle. Relinquishing treatment decision-making to a trusted partner at this emotionally difficult time may reduce the pressure on the woman on the one hand, but is also a precarious move on the other, especially if the husband does not share his wife’s views or knowledge about the treatment. Thus, among couples where the wife has chosen to hand over PS and DM, there could be a high level of consensus and pre-established trust in fertility-related issues, which are impetuses for harmonious relationships. Higher marital satisfaction may also reduce the woman’s distress and enable her to place greater trust in and be more open to suggestions from the healthcare team. Hence, a high level of marital satisfaction could be the antecedent for handing-over decision-making tasks to husbands and doctors, rather than its consequence. Fertility treatment decision-making epitomizes how marital and doctor-patient relationships interact and influence each other. Future studies are encouraged to examine the interactions of these relationships in a contextualized and dynamic manner.

### Limitations

In addition to self-selection bias in recruitment, this cross-sectional study provides only a snapshot of the experience of women in IVF treatment and cannot infer the direction of causality. Decision-making participation preferences could change with increasing knowledge, treatment experience, and relationships with other decision-makers such as doctors and a partner. Future studies should adopt a longitudinal approach to examine changes in participation preferences and clarify the antecedents and consequences of these changes. We also only included women with experience of a recent unsuccessful IVF cycle. Their decision-making participation preferences could be different from women who have not initiated treatment, are in active treatment or who have already terminated treatment. Lastly, this study investigated participation preferences from the vantage point of the women rather than actual participation of the women, their partners and doctors. Future studies should investigate the perspectives of partners and doctors and develop means to improve the congruence of actual and preferred participation of all parties.

### Practice implications

Despite the complexity of treatment decisions, our findings highlight that in partnership with doctors, women were keen to find solutions to their fertility problems as well as weighing various treatment options to arrive at a decision they deemed the best for them and their families. Echoing European Society of Human Reproduction and Embryology (ESHRE) guidelines [36] on psychosocial care in fertility treatment, our findings underscore the importance of providing information and decisional support to patients before, during and after a fertility treatment cycle. Not only is factual information about the pros, the cons and what to expect from different treatment and non-treatment options (e.g., adoption) important, decisional support in weighing different factors in relation to the unique situation of the woman and relational dynamics is also vital. The ultimate decision in fertility treatment is usually a trade-off among multiple factors that tend to be rather idiosyncratic and sometimes contradictory, including physical burden, psychological distress, social and familial expectations, desires for a biological child, financial affordability, etc. [5, 37]. A previous German study found that fertility patients were not well equipped to make informed treatment decisions because of their overwhelming desire for a child and insufficient information about the psycho-social-economic costs of treatment [5]. Counsellors should pay particular attention to these tangible and intangible costs and desires, screen for psychological and relational distress using validated measures and offer appropriate emotional and decisional support to couples throughout their treatment journey.

Unlike many other health conditions fertility treatment is marked by its relational nature [19]. Our findings highlight the significance of husbands’ involvement in decision-making from the viewpoint of their wives, and the associations between participation preferences and marital satisfaction. Chinese couples often face enormous stigma for being childless from both paternal and maternal families [24]. A husband’s involvement has been found to be pivotal both for his wife’s and his own adjustment [23, 38]. However, most husbands feel alienated in fertility treatment as many procedures and decisions concern their wife only [35]. Men are often involved in a typical IVF cycle at two points only – consenting to the treatment and providing a semen sample. Previous studies found that husbands tend to perceive themselves as a stoic “emotional rock” to support their wife, an agent exercising a “rational veto” and responsibility, and/ or a “biological necessity” to provide semen [20, 39, 40]. The supportive role aside, the mere fact of infertility could be emasculating [41]. Guilt is commonly experienced, especially when witnessing the physical and emotional duress experienced by their partner because of their shared desire for a biological child [22], and is particularly salient in cases of male-factor infertility [42]. The prospect of involuntary childlessness is daunting for many men who have long aspired to be a father [43]. Under such threats to virility, the pressure to be strong and masculine escalates, making disclosure of distress and help-seeking even harder [44–46]. Hence, patient enablement and counselling in fertility settings should include husbands whenever appropriate and possible. To start with, healthcare professionals should acknowledge the construction of treatment preference as a multi-factorial and dynamic interplay between intuitive and deliberative mental processes of both the woman and her partner. To achieve a couple-oriented approach, healthcare professionals should ensure husbands are offered adequate emotional, informational, and decisional support in fertility treatment. Fertility treatment has long been positioned as a feminine discipline. Nonetheless, future research should examine how much and in what ways husbands expect to be engaged in fertility treatment and its decision-making, as well as their understanding of infertility, desire for fatherhood and experiences in ART (e.g., sperm extraction, sperm donation, etc). The knowledge generated by this study will build the evidence-base for gender-sensitive and couple-oriented psychosocial support.

## Conclusion

Decision-making in fertility treatment is characterized by a unique interplay between a woman, her doctor, and her partner. This study examined treatment decision-making participation preferences among women with a recent unsuccessful IVF cycle. Our findings reveal the urge among women undergoing IVF to share treatment decision-making tasks with their doctors and actively involve their partner in the process.

## Supplementary information

**Additional file 1.** Questionnaire scales in English. Complete questionnaire scales used in this study in English.

## Data Availability

The datasets used and/or analysed during the current study are available from the corresponding author on reasonable request.

## Abbreviations

ART: Assisted reproductive technologies
CI: Confidence interval
DM: Decision-making
ESHRE: European Society of Human Reproduction and Embryology
FertiQOL: Fertility-related Quality of life
HADS: Hospital Anxiety and Depression Scale
IVF: In vitro fertilization
KMSS: Kansas Marital Satisfaction Scale
OR: Odds ratio
PS: Problem-solving
PSDM: Problem Solving-Decision Making
