# Supplementary material for: Preferred problem solving and decision-making role in fertility treatment among women following an unsuccessful in vitro fertilization cycle
Source: BMC Womens Health. 2019 Dec 5;19:153. doi: 10.1186/s12905-019-0856-5 (PMC6896772; doi:10.1186/s12905-019-0856-5)
Supplement: Supplementary file 1 — Additional file 1. Questionnaire scales in English. Complete questionnaire scales used in this study in English. [file 12905_2019_856_MOESM1_ESM.pdf]

Reference No.:___________ Date: ___________ (dd/mm/yyyy)

Co-organized by the

Department of Social Work and Social Administration and

Department of Obstetrics and Gynaecology, HKU

Treatment decision-making among women in IVF

Psychosocial evaluation form

Questionnaire (1)

Thank you for your participation!

With the help of this study, we hope to look into the psychosocial well-being of women undergoing IVF treatment, and in doing so, lay the foundation for future intervention work. Your participation will provide valuable insights to our understanding of the medical and psychological support needs of other women in IVF. Please try your best to answer all questions fully and accurately.

Respondent details

1. Name ______________ (Eng) ______________ (Chin)

2. Date of birth ______________ (dd/mm/yyyy)

3. Contact number ______________

4. Email address ____________________________

5. Date of marriage ______________ (dd/mm/yyyy)

6. Age of husband ______________ years

7. Are you receiving psychiatric treatment? ☐ Yes ☐ No

(If 'Yes', please indicate diagnosis and year of onset:___________________________________)

8. Are you taking psychiatric medications? ☐ Yes ☐ No

9. Education ☐ No formal education ☐ Primary level ☐ Secondary level ☐ Tertiary level or above ☐ Others: ________________________

10. Religion ☐ Nil ☐ Traditional Chinese beliefs ☐ Catholicism ☐ Christianity ☐ Buddhism ☐ Others:_________________

11. Employment ☐ Half-time ☐ Full-time ☐ Unemployed ☐ Others: ____

12. Do you and your husband have children? ☐ Yes ☐ No

(If 'Yes', number of children:____, via: *natural conception/assisted reproduction/adoption)

13. How long have you been trying to get pregnant? _____________ months

14. In which year have you had the first assisted reproductive technology (ART) cycle? ___________

15.

16. What is your ideal number of children in a family? :____ (Boy:____, Girl:____)

17. Below are questions about your fertility problem (i.e. infertility):

(a) Have you and your partner underwent ART treatment before now? ☐ Yes ☐ No

(b) If 'Yes', which type of ART treatment have you and your partner received?

	✓	Times		✓	Times
Ovulation induction (OI)			Intrauterine insemination (IUI)		
In vitro fertilization / Embryo transfer (IVF/ET)			Intracytoplasmic sperm injection (ICSI)		
Embryo donation			Egg donation		
Sperm donation			Others:___________________		

(c) Have you and your partner had a successful pregnancy via ART? ☐ Yes ☐ No

(d) Have you and your partner gave birth to a child via ART? ☐ Yes ☐ No

18. Below are questions about reproductive loss:

(a) Have you had experience of reproductive loss (e.g. miscarriage, stillbirth?) ☐ Yes ☐ No

(b) If 'Yes', which type of reproductive loss have you experiences?

(If more than once, please indicate the year in which they happen, duration and weeks of pregnancy)

☐ Artificial abortion (how many times:____; happening year:____; ____ weeks pregnant)

☐ Accidental abortion (how many times:____; happening year:____; ____ weeks pregnant)

☐ Others, please specify: ____

1. Please read carefully the below statements, and indicate the extent to which these statements reflect what you feel in the past week by ticking in the corresponding box. Answer according to your most immediate thought, your instinct is almost always more accurate than a pondered response

1. I feel tense or 'wound up'
   - ☐ Most of the time
   - ☐ A lot of the time
   - ☐ From time to time, occasionally
   - ☐ Not at all
2. I still enjoy the things I used to enjoy
   - ☐ Definitely as much
   - ☐ Not quite so much
   - ☐ Only a little
   - ☐ Hardly at all
3. I get a sort of frightened feeling as if something awful is about to happen
   - ☐ Very definitely and quite badly
   - ☐ Yes, but not too badly
   - ☐ A little, but it doesn't worry me
   - ☐ Not at all
4. I can laugh and see the funny side of things
   - ☐ As much as I always could
   - ☐ Not quite so much now
   - ☐ Definitely not so much now
   - ☐ Not at all
5. Worrying thoughts go through my mind
   - ☐ A great deal of the time
   - ☐ A lot of the time
   - ☐ From time to time, but not too often
   - ☐ Only occasionally
6. I feel cheerful:
   - ☐ Not at all
   - ☐ Not often
   - ☐ Sometimes
   - ☐ Most of the time
7. I can sit at ease and feel relaxed:
   - ☐ Definitely
   - ☐ Usually
   - ☐ Not often
   - ☐ Not at all
8. I feel as if I am slowed down
   - ☐ Nearly all the time
   - ☐ Very often
   - ☐ Sometimes
   - ☐ Not at all
9. I get a sort of frightened feeling like 'butterflies' in the stomach
   - ☐ Nearly all the time
   - ☐ Very often
   - ☐ Sometimes
   - ☐ Not at all
10. I have lost interest in my appearance:
    - ☐ Definitely
    - ☐ I don't take as much care as I should
    - ☐ I may not take quite as much care
    - ☐ I take just as much care as ever
11. I feel restless as I have to be on the move
    - ☐ Very much indeed
    - ☐ Quite a lot
    - ☐ Not very much
    - ☐ Not at all
12. I look forward with enjoyment to things
    - ☐ As much as I ever did
    - ☐ Rather less than I used to
    - ☐ Definitely
    - ☐ Hardly at all
13. I get sudden feelings of panic
    - ☐ Very often indeed
    - ☐ Quite often
    - ☐ Not very often
    - ☐ Not at all
14. I can enjoy a good book or radio or TV program:
    - ☐ Often
    - ☐ Sometimes
    - ☐ Not often
    - ☐ Very seldom

2. Please read carefully the below statements and circle the numbers that best reflect what you think about an ideal marital relationship.

   1=Extremely dissatisfied; 2=Very dissatisfied; 3=Slightly dissatisfied; 4=Neither satisfied or dissatisfied; 5=Slightly satisfied; 6=Very satisfied; 7= Extremely satisfied

1.	How satisfied are you with your marriage?	1	2	3	4	5	6	7
2.	How satisfied are you with your husband as a partner?	1	2	3	4	5	6	7
3.	How satisfied are you with the relationship between yourself and your partner?	1	2	3	4	5	6	7

3. (A) For each question, kindly check (tick the box) for the response that most closely reflects how you think and feel. Relate your answers to your current thoughts and feelings. Some questions may relate to your private life, but they are necessary to adequately measure all aspects of your life.

		Very Poor	Poor	Neither Good nor Poor	Good	Very Good
A.	How would you rate your health?	☐	☐	☐	☐	☐
		Very Dissatisfied	Dissatisfied	Neither Satisfied Nor Dissatisfied	Satisfied	Very Satisfied
B.	Are you satisfied with your quality of life?	☐	☐	☐	☐	☐
		Completely	A Great Deal	Moderately	Not Much	Not At All
1.	Are your attention and concentration impaired by thoughts of infertility?	☐	☐	☐	☐	☐
2.	Do you think you cannot move ahead with other life goals and plans because of fertility problems?	☐	☐	☐	☐	☐
3.	Do you feel drained or worn out because of fertility problems?	☐	☐	☐	☐	☐
4.	Do you feel able to cope with your fertility problems?	☐	☐	☐	☐	☐
		Very Dissatisfied	Dissatisfied	Neither Satisfied Nor Dissatisfied	Satisfied	Very Satisfied
5.	Are you satisfied with the support you receive from friends with regard to your fertility problems?	☐	☐	☐	☐	☐
6.	Are you satisfied with your sexual relationship even though you have fertility problems?	☐	☐	☐	☐	☐
		Always	Very Often	Quite Often	Seldom	Never
7.	Do your fertility problems cause feelings of jealousy and resentment?	☐	☐	☐	☐	☐

8. Do you experience grief and/or feelings of loss about not being able to have a child (or more children)?	☐	☐	☐	☐	☐
	Always	Very Often	Quite Often	Seldom	Never
9. Do you fluctuate between hope and despair because of fertility problems?	☐	☐	☐	☐	☐
10. Are you socially isolated because of fertility problems?	☐	☐	☐	☐	☐
11. Are you and your partner affectionate with each other even though you have fertility problems?	☐	☐	☐	☐	☐
12. Do your fertility problems interfere with your day-to-day work or obligations?	☐	☐	☐	☐	☐
13. Do you feel uncomfortable attending social situations like holidays and celebrations because of your fertility problems?	☐	☐	☐	☐	☐
14. Do you feel your family can understand what you are going through?	☐	☐	☐	☐	☐
	An Extreme Amount	Very Much	A Moderate Amount	A Little	Not At All
15. Have fertility problems strengthened your commitment to your partner?	☐	☐	☐	☐	☐
16. Do you feel sad and depressed about your fertility problems?	☐	☐	☐	☐	☐
17. Do your fertility problems make you inferior to people with children?	☐	☐	☐	☐	☐
18. Are you bothered by fatigue because of fertility problems?	☐	☐	☐	☐	☐
19. Have fertility problems had a negative impact on your relationship with your partner?	☐	☐	☐	☐	☐
20. Do you find it difficult to talk to your partner about your feelings related to infertility?	☐	☐	☐	☐	☐
21. Are you content with your relationship even though you have fertility problems?	☐	☐	☐	☐	☐
22. Do you feel social pressure on you to have (or have more) children?	☐	☐	☐	☐	☐
23. Do your fertility problems make you angry?	☐	☐	☐	☐	☐
24. Do you feel pain and physical discomfort because of your fertility problems?	☐	☐	☐	☐	☐

(B) Have you started fertility treatment (this includes any medical consultation or intervention)? If Yes, then please respond to the following questions. For each question, kindly check (tick the box) for the response that most closely reflects how you think and feel. Relate your answers to your current thoughts and feelings. Some questions may relate to your private life, but they are necessary to adequately measure all aspects of your life.

		Always	Very Often	Quite Often	Seldom	Never
1.	Does infertility treatment negatively affect your mood?	☐	☐	☐	☐	☐
2.	Are the fertility medical services you would like available to you?	☐	☐	☐	☐	☐
		An Extreme Amount	Very Much	A Moderate Amount	A Little	Not At All
3.	How complicated is dealing with the procedure and/ or administration of medication for your infertility treatment(s)?	☐	☐	☐	☐	☐
4.	Are you bothered by the effect of treatment on your daily or work-related activities?	☐	☐	☐	☐	☐
5.	Do you feel the fertility staff understand what you are going through?	☐	☐	☐	☐	☐
6.	Are you bothered by the physical side effects of fertility medications and treatment?	☐	☐	☐	☐	☐
		Very Dissatisfied	Dissatisfied	Neither Satisfied Nor Dissatisfied	Satisfied	Very Satisfied
7.	Are you satisfied with the quality of services available to you to address your emotional needs?	☐	☐	☐	☐	☐
8.	How would you rate the surgery and/or medical treatment(s) you have received?	☐	☐	☐	☐	☐
9.	How would you rate the quality of information you received about medication, surgery and/or medical treatment?	☐	☐	☐	☐	☐
10.	Are you satisfied with your interactions with fertility medical staff?	☐	☐	☐	☐	☐

4. The below questions will ask of your preference in medical decision-making.

(A) Suppose you had mild chest pain for three days and decided that you should visit your doctor about this –

	The doctor alone	Mostly the doctor	Both equally	Mostly me	Me alone
1.Who should determine (diagnose) what the likely causes of your symptoms are?	1	2	3	4	5
2.Who should determine what the treatment options are?	1	2	3	4	5
3.Who should determine what the risks and benefits for each treatment option are?	1	2	3	4	5
4.Who should determine how likely each of these risks and benefits are to happen?	1	2	3	4	5
5.Given the risks and benefits of these possible treatments, who should decide how acceptable those risks and benefits are for you?	1	2	3	4	5
6.Given all the information about risks and benefits of the possible treatments, who should decide what treatment option should be selected?	1	2	3	4	5

(B) Based on you and your partner's infertility at the moment, do you think –

1. Who should determine (diagnose) what the likely causes of your infertility are?

Doctor	Husband	Myself
No role at all / Shared / Should make all decisions	No role at all / Shared / Should make all decisions	No role at all / Shared / Should make all decisions
0 - 1 - 2 - 3 - 4	0 - 1 - 2 - 3 - 4	0 - 1 - 2 - 3 - 4

2. Who should determine what the treatment options are?

Doctor	Husband	Myself
No role at all / Shared / Should make all decisions	No role at all / Shared / Should make all decisions	No role at all / Shared / Should make all decisions
0 - 1 - 2 - 3 - 4	0 - 1 - 2 - 3 - 4	0 - 1 - 2 - 3 - 4

3. Who should determine what the risks and benefits for each treatment option are?

Doctor	Husband	Myself
No role at all / Shared / Should make all decisions	No role at all / Shared / Should make all decisions	No role at all / Shared / Should make all decisions
0 - 1 - 2 - 3 - 4	0 - 1 - 2 - 3 - 4	0 - 1 - 2 - 3 - 4

4. Who should determine how likely each of these risks and benefits are to happen?

Doctor	Husband	Myself
No role at all / Shared / Should make all decisions	No role at all / Shared / Should make all decisions	No role at all / Shared / Should make all decisions
0 - 1 - 2 - 3 - 4	0 - 1 - 2 - 3 - 4	0 - 1 - 2 - 3 - 4

5. Given the risks and benefits of these possible treatments, who should decide how acceptable those risks and benefits are for you?

Doctor	Husband	Myself
No role at all / Shared / Should make all decisions	No role at all / Shared / Should make all decisions	No role at all / Shared / Should make all decisions
0 - 1 - 2 - 3 - 4	0 - 1 - 2 - 3 - 4	0 - 1 - 2 - 3 - 4

6. Given all the information about risks and benefits of the possible treatments, who should decide what treatment option should be selected?

Doctor	Husband	Myself
No role at all / Shared / Should make all decisions	No role at all / Shared / Should make all decisions	No role at all / Shared / Should make all decisions
0 - 1 - 2 - 3 - 4	0 - 1 - 2 - 3 - 4	0 - 1 - 2 - 3 - 4

5. This section asks about your thoughts on children and childbearing, please indicate the extent which you agree with each statement. There is no right or wrong answer, we would like to know your thoughts.

	Strongly disagree	Disagree	Neutral	Agree	Strongly agree
1. To continue on the family line is part of the responsibility after marriage	☐	☐	☐	☐	☐
2. I believe in the saying that to raise a child is to protect oneself against old age	☐	☐	☐	☐	☐
3. I believe not having children would be my biggest failure as a daughter	☐	☐	☐	☐	☐
4. Without children, I feel I have not fulfilled my responsibility as daughter-in-law and have failed my parents-in-law	☐	☐	☐	☐	☐
5. I would like my parents to feel the happiness of having grandchildren	☐	☐	☐	☐	☐
6. Without children, I feel I have not fulfilled my responsibility as a wife and have failed my husband	☐	☐	☐	☐	☐

6. You are asked to indicate your thoughts on infertility treatment:

1.	Very unimportant									Very important
In general, to me, childbearing is...	1	2	3	4	5	6	7	8	9	10
In general, to my partner, childbearing is...	1	2	3	4	5	6	7	8	9	10
In general, to my marital relationship, childbearing is...	1	2	3	4	5	6	7	8	9	10
In general, to my family, childbearing is...	1	2	3	4	5	6	7	8	9	10

2. Please rank the following five possible treatment outcomes from IVF using 1,2, 3, 4, and 5 in order of preference (1 = most preferred, 5 = least preferred)

No child _____

One child (singleton pregnancy) _____

Two children (Twin pregnancy) _____

Three children (Triplet pregnancy) _____

Four or more children (quadruplets or more) _____

3. If you ranked a singleton pregnancy above a twin in question 2, move on to question 4. If you desire a multiple gestation over a singleton, please rate the following reason using 1, 2, 3, 4, and 5, in order of importance (1 = most important, 5 = least important)

I want to reach my ideal family size more quickly _____

I do not have the finances to afford more infertility treatments in the future _____

Twins run in my family _____

I do not want the stress and bother of IVF again in the future _____

Others (please list): _____

4. Below questions concern knowledge about twins. If you are unsure about the answer, please select what you think is the more probable answer.

(a) A full term delivery is at 40 weeks gestation age, the average twin pregnancy delivers at:

☐ 32 weeks ☐ 34 weeks ☐ 36 weeks ☐ 38 weeks ☐ 40 weeks

Is the below scenarios more likely to happen in singleton pregnancy or twin pregnancy?

(b) Pre-term delivery ☐ Singleton pregnancy ☐ Twin pregnancy ☐ Same for both

(c) Stillborn ☐ Singleton pregnancy ☐ Twin pregnancy ☐ Same for both

(d) Dying in the first year of life ☐ Singleton pregnancy ☐ Twin pregnancy ☐ Same for both

(e) Born with cerebral palsy ☐ Singleton pregnancy ☐ Twin pregnancy ☐ Same for both

(f) Body malformation ☐ Singleton pregnancy ☐ Twin pregnancy ☐ Same for both

(g) High blood pressure in mother ☐ Singleton pregnancy ☐ Twin pregnancy ☐ Same for both

(h) Mother has heavy bleeding ☐ Singleton pregnancy ☐ Twin pregnancy ☐ Same for both

5. How many embryos did you transfer in the last treatment cycle? ☐ One ☐ Two

How stressed/distressed so you feel in the last treatment cycle?

	Not stressed at all										Extremely stressed
Physical discomfort brought about by treatment	0	1	2	3	4	5	6	7	8	9	10
Psychological distress brought about by treatment	0	1	2	3	4	5	6	7	8	9	10
Conflict with partner/family brought about by treatment	0	1	2	3	4	5	6	7	8	9	10
Frustration about life/work brought about by treatment	0	1	2	3	4	5	6	7	8	9	10
Financial burden brought about by treatment	0	1	2	3	4	5	6	7	8	9	10
Overall, the level of distress experienced in treatment	0	1	2	3	4	5	6	7	8	9	10

6. To your knowledge, in general, what is the success rate of an IVF treatment cycle? (from 0 to 100)

One embryo: ________________% Two embryos: ________________%

To your knowledge, in general, what is rate of having twin pregnancy from an IVF treatment cycle?

One embryo: ________________% (note: it is still possible that one embryo will split into two)

Two embryos: ________________%

7. Your next treatment cycle will be: ◻ Embryo transfer ◻ Ovulation induction + embryo transfer

What do you think the success rate of your next treatment will be? (from 0 to 100) ________________%

At this moment, do you think you will undergo a new treatment cycle?

☐ Definitely ☐ Likely ☐ Half/ half ☐ Unlikely ☐ Definitely not

8. How important is each of the below factors for you when deciding whether or not to continue treatment?

	Very unimportant	Rather Unimportant	Neutral	Rather important	Very important
The safety of the baby	0	1	2	3	4
The safety of the mother	0	1	2	3	4
Rate of successful pregnancy	0	1	2	3	4
Your own's preferences on childbearing	0	1	2	3	4
Your husband's preferences on childbearing	0	1	2	3	4
Your family's preferences on childbearing	0	1	2	3	4
Physical discomfort from treatment	0	1	2	3	4
Psychological distress from treatment	0	1	2	3	4
Conflicts with partner/family because of treatment	0	1	2	3	4
Frustration about life/work brought about by treatment	0	1	2	3	4
Financial burden brought about by treatment	0	1	2	3	4
Doctor's suggestion	0	1	2	3	4

\- End of the questionnaire -
